# Transition of care in a Danish context: translation, cross-cultural adaptation and content validation of CTM-15 and PACT-M

**DOI:** 10.1186/s41687-024-00739-3

**Published:** 2024-06-10

**Authors:** Merete Ajstrup, Caroline Trillingsgaard Mejdahl, David Høyrup Christiansen, Lene Kongsgaard Nielsen

**Affiliations:** 1 Research Unit for Multimorbidity, Department of Cardiology, Viborg Regional Hospital, Heibergs Allé 2K, Viborg, 8800 Denmark; 2 Centre for Research in Health and Nursing, Viborg Regional Hospital, Heibergs Allé 2K, Viborg, 8800 Denmark; 3 DEFACTUM—Public Health Research, Olof Palmes Alle 15, Aarhus, 8200 Denmark; 4 Department of Clinical Medicine, Health, Aarhus University, Palle Juul-Jensens Blvd. 82, Aarhus, 8200 Denmark; 5 University Clinic, Elective Surgery Centre, Silkeborg Regional Hospital, Falkevej 1A, Silkeborg, 8600 Denmark; 6 Quality of Life Research Center, Odense University Hospital, Odense, Denmark; 7 Department of Internal Medicine, Gødstrup Hospital, Herning, Denmark

**Keywords:** Patient reported experience measure, Patient reported outcome, Content validity, Multimorbidity, Discharge

## Abstract

**Background:**

Transition of care from hospitalisation to home is a complex process with potential patient safety risks, especially for patients with multimorbidity. Traditionally, the quality of transition of care has been evaluated primarily through readmission rates. However, interpreting the readmission rates presents challenges, and readmission rates fail to capture the patient’s perspective on the quality of the care transition. Insight into the patient’s experience with their care or a health service can be provided through the use of patient-reported experience measures (PREMs), and the two PREMs Care Transitions Measure 15 (CTM-15) and Partners at Care Transitions Measure part 1 and 2 (PACT-M1 and PACT-M2) assess on the quality of transition of care from the patients’ perspective. The aim of this study was to translate, culturally adapt, and assess content validity of CTM-15, PACT-M1, and PACT-M2 for Danish-speaking patients with multimorbidity.

**Methods:**

A two-step approach was used for content validation, involving cognitive debriefing and interviews with patients, representing the target group, as well as quantitative data collection from healthcare professionals representing all three sectors of the Danish healthcare system. The patients were systematically interviewed regarding the aspects of content validity; comprehensibility, relevance, and comprehensiveness. The healthcare professionals assessed the relevance and comprehensiveness of each item through questionnaires, allowing the calculation of a content validity index (CVI). An item CVI ≥ 0.78 is considered good.

**Results:**

The results of the qualitative data indicated that both CTM-15 and the PACT-M questionnaires were considered relevant, and comprehensible, and comprehensive to the target group. The CVI computed at item level determined that PACT-M1 and PACT-M2 demonstrated excellent content validity among the healthcare professionals, whereas the CVI for two items of the CTM-15 fell below the threshold value for “good”.

**Conclusion:**

The Danish versions of the PACT-M questionnaires demonstrated good content validity, and the CTM-15 demonstrated acceptable content validity based on qualitative data from patients and quantitative data from healthcare professionals. Further validation of the questionnaires, by assessing their construct validity and reliability is recommended.

## Abstract (plain language summary)

In this study, we aimed to translate two questionnaires, CTM-15 and PACT-M, that address patients’ perspectives on the quality of transition of care, into Danish. Furthermore, we assessed their content validity i.e., their relevance, comprehensibility, and comprehensiveness. The study is important because we need to include the patients’ experiences in the overall evaluation of transition of care from hospital admission to home. This is possible through validated questionnaires. In order to investigate the content validity, it is important to include representatives of the target group, in this study patients with multimorbidity, as well as professional experts in the area. We interviewed patients about their perception of the relevance, comprehensibility, and comprehensiveness of the questionnaires. Additionally, we asked healthcare professionals about the relevance and comprehensiveness of the questionnaires. PACT-M demonstrated good content validity according to both patients and healthcare professionals. CTM-15 showed good content validity among patients, however, two items were not considered relevant by healthcare professionals. To ensure the usability of the questionnaires, further investigation into their construct validity, reliability, and responsiveness is necessary. Upon completion of the validation process, the questionnaires will be valuable tools in research projects aiming to improve the transition of care, as well as in quality monitoring and improvement initiatives.

## Background

Transitions of care between hospitals and primary care settings are recognised as high risk scenarios for patient safety [1]. The process of transition of care from hospitalisation to home is a complex and multifaceted process with potential risks such as medication errors, insufficient care coordination, patient and caregiver uncertainty, heightened healthcare utilisation, and preventable readmissions [1–3]. Patients with multimorbidity, defined as living with two or more chronic conditions, are particularly susceptible to experiencing one or more of these adverse events [4–7].

Transitions of care are an integral part of a patient’s journey throughout a healthcare system, and refer to the various points where a patient moves to, or returns from, a particular physical location or makes contact with a healthcare professional for the purposes of receiving health care [1]. Transition of care can be defined as a set of actions designed to ensure the coordination and continuity of healthcare services as patients transfer between different locations or different levels of care within the same location [8].

To enhance transition of care and mitigate the risk of adverse events, it becomes essential to conduct a comprehensive and nuanced evaluation of the quality of these transitions.

Traditionally, the quality of transition of care has been gauged primarily through readmission rates [9, 10]. However, interpreting readmission rates presents challenges [11]. Relying solely on readmission rates hinder the ability to pinpoint specific aspects of the transition process requiring improvement, as these rates are influenced by the actions of various healthcare providers and factors related to the individual patient, including their primary medical condition, age, co-morbidities, health literacy, and social circumstances [9–12]. Moreover, readmission rates fail to capture the patient’s perspective on the quality of the care transition.

Evidence across different areas of healthcare indicates that patient experience is clinically important in investigating the quality in healthcare, alongside clinical effectiveness and patient safety [13]. Given the organisational fragmentation of much of healthcare and the numerous services with which many patients interact, the measurement of patient experience may help provide a ‘whole-system’ perspective [13].

Patient experience holds considerable importance in comprehending healthcare quality, and patient experience data, robustly collected and analysed, can shed light on both strengths and weaknesses in terms of effectiveness and safety [13]. There is increasing international attention regarding the use of patient-reported experience measures (PREMs) as a quality indicator of patient care and safety, and insight into the patient’s experience with their care or a health service, can be provided through the use of PREMs [14].

In a systematic review, seven measurement tools that assess transitional care quality from the patient’s perspective have been identified and appraised [15]. Among these, Care Transitions Measure-15 (CTM-15) and PACT-M are the most comprehensive in covering the transition of care process [16–19].

The CTM-15 and PACT-M have been translated and validated in several countries [20, 21]. However, whether these measures can assess the quality and safety of transitional care for patients with multimorbidity in Denmark has not been investigated. Therefore, the aims of this study were to translate and cross-culturally adapt the CTM-15 and the PACT-M questionnaires for use in a Danish-speaking population. Moreover, to assess and compare content validity of the two questionnaires in terms of relevance, comprehensibility and comprehensiveness among patients with multimorbidity, and among healthcare professionals.

## Methods

### Instruments

#### CTM-15

The original CTM-15 was developed in the USA in 2003 to assess the quality of care transitions across healthcare settings and to provide a better understanding of the care transition experiences from the patient’s perspective [17]. The objective was to create a metric that aligns with the principles of patient-centeredness, both in substance and methodology, and can be effectively utilized for performance measurement and public reporting [16]. The CTM-15 consists of four sections covering *preferences taking into account at the hospital*, *preparations to leave the hospital*, *preparations on follow-up arrangements*, and *medication management*.

#### PACT-M

The PACT-M was developed in the UK in 2019 with the aim of creating a tool to evaluate patient experiences during care transitions. Its purpose is to serve as an indicator of transitional care quality by assessing the patients’ experience of the transition process [18]. PACT-M covers both the immediate post-discharge period and the longer-term experience of managing health and care at home by examining the patients’ experience at two points of time; one week after discharge (using PACT-M1) and one month after discharge (using PACT-M2) [15].

The PACT-M comprehensively addresses eight components related to patients’ transitional care experience from hospital to home; *patient involvement*, *medication management*, *discharge arrangements*, *coordination with other providers*, *providing information and guidance to patient/family*, *providing psychological and social support*, *anticipation and preparation for emergencies/deterioration*, and *feeling safe* [18]. Furthermore, it incorporates a list of seven questions to capture potential care-related problems and adverse events commonly encountered by recently discharged patients [19].

### Participants and setting

The study was conducted in Denmark at four wards at a medium sized hospital in Central Denmark Region. The study was carried out from November 2022 to March 2023.

#### Patients and inclusion procedure

We aimed to recruit patients who represented demographic diversity in terms of gender, age, and marital status to enhance the likelihood that the findings would be valuable and comprehensive [22, 23]. A priori, it was decided to interview at least seven patients, which is considered an appropriate number of patients according to the COSMIN Methodology [24].

The inclusion criteria for the study encompassed: multimorbidity, defined as living with two or more chronic conditions [7], individuals aged 18 years or older, patients who were planned to be discharged to their homes after being hospitalised for an acute medical condition, and finally, patients should be native speakers of the Danish language. Exclusion criteria were: Patients with mental disorders that impede their ability to understand and respond appropriately to the questions in the provided questionnaires, and individuals who were incapable of providing written and oral informed consent.

Eligible patients were identified by the treating clinicians in the ward. The clinician introduced the patients to the project. Patients who agreed to learn more about the project were contacted by MA and were informed about the project orally and in writing. Only patients, who provided oral and written consent for participation, were included in the project. Information about their age, education level, and cohabitation status was obtained. Information regarding the patient’s chronic conditions was obtained through a combination of patient inquiry and chart review. Furthermore, the patient’s score on the clinical frailty scale was assessed [25, 26]. The same inclusion and exclusion criteria were used for patient expert panel I and II.

#### Healthcare professionals

We included healthcare professionals from two different professions, medical doctors and nurses, from the three sectors of the Danish healthcare system; the hospital, the municipality, and general practitioners. To ensure the best possible evaluation, all healthcare professionals included in the study had experience within patients with multimorbidity and transition of care [24, 27].

### Translation procedure

The translation procedure followed the ISPOR principles of Good Practice for the Translation and Cultural Adaption Process for Patient-Reported Outcomes (PRO) Measures in combination with the EORTC Quality of Life Group Translation Procedure [28, 29].

The process included the following steps: forward translation, reconciliation, back translation, back translation review, pre-testing, review of cognitive debriefing results and finalisation, proof reading, and final report of translation, including the final versions of the questionnaires [28, 29].

Before the translation, the instrument developers were contacted and granted permission for the translation of the questionnaires to Danish versions. The developer of CTM-15 did not participate in the process. The developers of PACT-M contributed to the process by addressing any uncertainties and providing additional considerations. In the forward translation, two native Danish speakers, one professional translator with experience within translating questionnaires, and one healthcare professional (MA), separately translated the English versions into Danish. The Danish versions were compared and reconciled into one by three native Danish speakers (the professional translator, LKN and MA). The translated versions were sent for back translation to two native speakers of English who had no knowledge of the original versions of the questionnaires. The back translations were reviewed and compared with the original version by LKN and MA, and the back translation of PACT-M was approved by the developers.

The pre-testing was conducted with patient expert panel I, who were all native speakers of Danish, and consisted of two parts:

1. Patients received and completed the three translated questionnaires
2. Debriefing. A researcher discussed the translation with the patients individually.

The debriefing was conducted in Danish, and the interviewing researcher was a native speaker of Danish. During the debriefing, the interviewing researcher (MA) went through each item of the questionnaires, asking the patient whether the translation was difficult to answer, confusing, difficult to understand or offensive. If the patient had any comments, the patient was invited to reword the question in a way that would be easier to understand, less confusing or less offensive. All comments were recorded on patient report sheets, and all comments were included in the review of the cognitive debriefing results.

Based on the cognitive debriefing results, final versions of the questionnaires were formulated by MA, CTM and LKN. MA and LKN proofread the questionnaires, and MA completed the final translation report.

### Content validity assessment

The assessment of content validity followed the COSMIN methodology for assessing the content validity of PROMs [24]. Content validity is the degree to which the content of an instrument is an adequate reflection of the construct to be measured [30]. The construct in this study being “transition of care”. Content validity refers to the relevance, comprehensiveness, and comprehensibility of the instrument for the construct, target population, and context of use. A first aspect is face validity, which was assessed by MA, LKN, and DHC [31]. Content validity was assessed by asking patients and healthcare professionals about the relevance, comprehensiveness and comprehensibility of the items, response options, and instructions [31].

The content validity from the patient’s perspectives was investigated through qualitative methods in order to focus on the patient’s voice and reach a more in-depth insight of the patient’s thoughts and experiences with the content of the instruments [32].

The content validity among healthcare professionals was investigated through quantitative methods, which allowed for a more systematic and standardised evaluation of content validity, in order to be able to test the content validity index of the questionnaires.

Part 1 of both PACT-M questionnaires consists of seven questions to capture potential care-related problems and adverse events. These were not included in the assessment of the quality of the care transition itself. Therefore, we chose to pre-test these questions to ensure their comprehensibility, but we did not include them in the actual content validation process as they do not directly relate to the underlying construct, but rather pertain to actual events.

#### Content validity—patients

*Procedure*

To assess the content validity of the final Danish questionnaires from the patients’ perspective, cognitive interviewing was performed on a new patient group (patient expert panel II). The cognitive interviews were performed to uncover potential problems with encoding or retrieval of information [32]. The interview technique was retrospective probing where the probes were administered once all the survey questions had been answered [33]. A semi-structured interview guide was formulated by CTM, LKN and MA allowing for a more dynamic and responsive conversation while ensuring that essential topics were covered. The interview guide was based on the concepts of the COSMIN methodology for assessing the content validity of PROMs, focusing on relevance and comprehensiveness [24]. The patients were asked whether each item was relevant for them, considering their own experience with transition of care. Furthermore, the patients were asked whether the questionnaire as a whole seemed meaningful and if any key concepts were missing, in order to assess whether the items together comprehensively cover the construct. To assess the overall comprehensibility, the patients were asked if they found the instructions, the response options, and the recall period or timing of the questionnaire appropriate. The comprehensibility of each item was addressed previously through the questions targeting the translation.

The cognitive interviewing consisted of two parts at three different points of time. Part 2 was carried out in continuation of part 1:

1. The patient received and completed the translated questionnaire
2. A researcher interviewed the patient about the relevance, comprehensiveness and comprehensibility of the questionnaire.

The cognitive interviews were carried out in the following points of time:

- CTM-15: at the day of discharge (T0)
- PACT-M1: within one week from discharge (T1)
- PACT-M2: within one month from discharge (T2).

The interviews were performed by MA, who were trained specifically for this study by an experienced qualitative researcher, CTM.

The interview at T0, was performed face-to-face with the patient at the ward before discharge. The cognitive interviews at T1 and T2 were planned to be performed at visits in the patient’s home, alternative at a location following the patients wish.

*Analysis of interviews*

All the interviews were recorded and transcribed verbatim. The qualitative data were managed in NVivo. Data from the interviews were analysed using a content analysis approach where content codes were sorted according to the aspects of content validity; relevance, comprehensiveness and comprehensibility [24]. All interview data were analysed after the last interview was conducted. The analyses were performed by MA and CTM with continuous input from and discussions with the research group.

The relevance, comprehensibility, and comprehensiveness of each questionnaire were assessed based on the analyses, thereby reflecting the content validity of the questionnaires from the patients’ perspective.

#### Content validity—healthcare professionals

*Procedure*

Questionnaires were administered electronically to 80 healthcare professionals from hospital, municipality, and general practice. If the questionnaire was not answered, a reminder was sent. A group of healthcare professionals at the hospital requested the possibility to answer the questionnaire on paper instead of electronically, which was accepted.

The healthcare professionals were asked about their job title and years of experience. They were then asked to read the CTM-15 and PACT-M carefully and subsequently to rate the relevance in terms of quality transition of care from hospital admission to home from the patients’ perspective of each item on a 4 point Likert scale from 1 (not relevant) to 4 (extremely relevant). Additionally, for each questionnaire they were asked whether they found any significant aspects to be missing.

*Data analysis*

To quantify the assessments of the healthcare professionals, the content validity index for each item (I-CVI) was computed. The I-CVI is the proportion of participants who rate the item as a 3 or 4 on the Likert scale. In addition, the average CVI (Ave-CVI), which represents the content validity of the overall instrument, was calculated by summing the I-CVIs for the instrument and dividing them by the number of items. An I-CVI ≥ 0.78 is considered good [34, 35], and the acceptable standard of Ave-CVI is 0.90 [34, 35].

## Results

### Face validity

MA, LKN and DHC assessed the face validity of the CTM-15 and PACT-M questionnaires as an overall view of the items, and established that the items seemed as an adequate reflection of the construct to be measured i.e., the quality of transition of care from the patients’ perspective.

### Translation and cultural adaption

The Danish versions of CTM-15 and PACT-M were pre-tested among patient expert panel I of 13 patients, who represented a variability of ages, gender, and educational levels (Table 1). All pre-testings were performed face-to-face at the hospital on the day of discharge.

**Table 1:** Characteristics, patient expert panel I

Factor	
Age (years), mean (SD)	68 (12)
Gender (female), n (%)	6 (46)
Score on clinical frailty scale, median (IQR)	3 (3;4)
Cohabitation status	
Living alone, n (%)	9 (69)
Living with spouse/cohabitant, n (%)	4 (31)
Education	
Short education or skilled worker, n (%)	10 (77)
Medium or long education, n (%)	3 (23)
Most prevalent diagnosis	High cholesterol Hypertension Prostate disorder Chronic pain condition Back disorders Arthrosis

*SD* standard deviation

*IQR* interquartile range

The cultural adaptations that were necessary are described below. The items presented in italics represent the English source item, while those not in italics are the English translation of the Danish revision.

#### CTM-15

Item 2 *“The hospital staff took my preferences and those of my family or caregiver into account in deciding what my health care needs would be when I left the hospital”.* Some patients expressed that the question was lengthy and somewhat confusing. It was challenging because the original item is complex, and we would like to end up with an item that closely resembled the original item. Therefore, we only made slight modifications to maintain the closest possible alignment with the original item and yet make it more comprehensible to the patients.

Item 12 *“When I left the hospital, I had a readable and easily understood written list of the appointments or tests I needed to complete within the next several weeks”.* In the Danish healthcare system, patients are not typically provided with written lists of future appointments. Furthermore, it is not uncommon to be discharged from the hospital with a follow-up appointment scheduled three months (or longer) in the future. Consequently, we did not find the time frame “the next several weeks” entirely appropriate. To better reflect the reality of the Danish healthcare system, we modified the wording to what we found aligned with the content of the original item: “When I left the hospital, I had a good overview of my future appointments and tests”.

#### PACT-M1

Part 1, item 2 *“Have you had any infections?”.* In Danish, the direct translation of “infection” is a term mostly used by healthcare professionals rather than by layman. During the debriefing some of the patients asked for examples of infections. Following correspondence with the developers, we incorporated “novel infections” along with examples of infections “such as urinary tract infection or pneumonia” in order to make the question more precise. The examples were chosen, as they are common and well-known layman terms.

Part 1, item 6 *“Have you had any problems getting essential healthcare supplies (like pads or prescribed feed)?”* posed a challenge in translation, as there is no exact equivalent for “healthcare supplies” in Danish. Furthermore, “pads” is not relevant to mention in a Danish healthcare context as patients have to buy pads themselves at the pharmacy. Therefore, in agreement with the developers, we added the word “aids” in the item, and included examples relevant in a Danish context to provide patients with a clearer understanding of the term’s meaning. The final Danish item was: “Have you had any problems getting essential aids or healthcare supplies (like a walker or prescription nutritional supplements)?”

Part 2, item 5 *“I understood how to get help or support from my community services (e.g. doctors, nurses, home care staff) if I needed it after returning home”*. We decided that “doctors” must be written outside the bracket, as they are not part of community services in a Danish context. The Danish version of the item being: “I understood how to get help or support from my general practitioner or my community services (e.g., nurses and homecare staff) if I needed it after returning home”.

#### PACT-M2

Part 2, item 2 *“I know how to manage my medicines”*. We decided to clarify *“manage my medicines”* with the specification “how to obtain my medication, when to take it, and the correct dosage”.

Part 2, item 3 *“I have the necessary support to manage everyday activities (e.g. cooking, cleaning, buying food, showering, walking, dressing)”.* Some of the patients in the debriefing, who did not receive homecare support, were requesting a “not applicable” response option. However, the developers recommended not to. Instead, they suggested to encourage patients, who did not need homecare support, to answer “strongly agree”. Accordingly, we added “If you do not need support, please mark ‘strongly agree’.”.

Part 2, item 5 *“I feel confident about managing my health at home”.* The Danish wording of the direct translation of “managing my health” was criticised by several patients in the debriefing. Therefore, we contacted the Danish Language Council to obtain a correct and at the same time more user-friendly wording of the item. All adaptions and additions for PACT-M were approved by the developers. The Danish version of each questionnaire was finalised and proofread, and a translation report of each step and each decision made was drafted.

### Content validity—patients

The cognitive interviews, which form the basis of the content validity assessment, were conducted among patient expert panel II including nine patients (Table 2). One of the interviews was conducted over the phone, all other interviews were conducted face-to-face, either in the hospital (at T0) or at the patient’s home (at T1 and T2).

**Table 2:** Characteristics, patient expert panel II

Factor	
Age (years), mean (SD)	67 (10)
Gender, female, n (%)	6 (67)
Score on clinical frailty scale, median (IQR)	4 (3;5.5)
Cohabitation status, n (%)	
Living alone, n (%)	2 (22)
Living with spouse/cohabitant, n (%)	7 (78)
Education	
Short education or skilled worker, n (%)	5 (56)
Medium or long education, n (%)	4 (44)
Most prevalent diagnosis	Chronic obstructive pulmonary disease High cholesterol Hypertension Psoriasis or eczema Hearing loss Back pain Arthrosis

*SD* standard deviation

*IQR* interquartile range

#### CTM-15

Overall, the patients found the items in the questionnaire to be relevant in assessing the transition of care from the patient’s perspective. A few patients found certain items to be irrelevant, while some suggested combining two or three questions into one (e.g., questions 13–15 about medication). The patients generally did not have any problems filling out the questionnaire.

The majority of the patients found the answer options appropriate, as the scale from strongly disagree to strongly agree was short and concise yet allowed for grading the response, and there was an option to select not applicable. However, most patients found the timing of the questionnaire challenging, as many items referred to activities that had not yet taken place or information not yet given at the time of the interview, as the respondents had not yet been discharged.

The patients found the items in the questionnaire to be comprehensive in describing their experience of transition of care. None of the respondents felt that there were areas of the transition of care that were not adequately covered in the questionnaire.

#### PACT-M1

Generally, the patients expressed that the items in the questionnaire were relevant in assessing the patient’s perspective on the transition of care. However, several patients who did not require homecare found the questions related to homecare to be irrelevant. Nonetheless, they also acknowledged the importance of including these questions as long as *“not applicable”* was an answer option. The patients found the questionnaire instructions to be comprehensive and easy to understand.

The majority of patients found the response options to be appropriate and useful in allowing them to indicate the extent to which they agreed or disagreed. Some patients requested a “don’t know/not applicable” option for all questions, as the agree/disagree scale may not be applicable to all respondents for all questions. This was particularly noted in questions regarding homecare. None of the patients expressed that the recall period was too long or in any way unsuitable for the statements in the questionnaire.

Additionally, the patients expressed that the questionnaire’s length and coverage of necessary aspects were adequate in describing the transition of care from the patient’s perspective.

#### PACT-M2

Several patients, who did not have the need for homecare, found that particularly item 3 “*I have the necessary support to manage everyday activities (e.g. cooking, cleaning, buying food, showering, walking, dressing)”*, which pertains to everyday support, was not relevant. Additionally, a few informants did not find item 8 “*I feel I can now manage my care safely at home”* relevant, as well. However, overall, the informants assessed the items as relevant for evaluating sector transitions from the patient’s perspective. The patients found the instructions for completing the questionnaire easy to understand.

The patients generally found the response options meaningful and appropriate for the items. Several patients indicated a need for a “not applicable” response category for statements regarding homecare. This was particularly mentioned for item number 3 “*I have the necessary support to manage everyday activities (e.g. cooking, cleaning, buying food, showering, walking, dressing)”* and was also noted for items 4 *“I feel I have the support I need from community health services (e.g. doctors, nurses, homecare staff)”* and 8 “*I feel I can now manage my care safely at home”*.

The majority of patients expressed that the items in the questionnaire were comprehensive, and there were no areas that were missing in order to assess the quality of transitions of care from the patients’ perspective.

### Content validity—healthcare professionals

In total, 57 healthcare professionals completed the questionnaires, of these, 10 completed on paper, assessing the content validity of CTM-15 and PACT-M. The healthcare professionals represented two different professions, nurses and medical doctors, and were from all three sectors of the Danish healthcare system. The characteristics of the healthcare professionals are presented in Table 3, and the CVIs of the CTM-15, PACT-M1 and PACT-M2 are presented in Table 4.

**Table 3:** Characteristics, healthcare professionals

	*N* (%)
Sector	
Hospital	42 (74)
Municipality	7 (12)
General practice	8 (14)
Job title	
Nurse	15 (26)
Medical doctor	42 (74)
Clinical experience (years)	
≤ 10	10 (18)
> 10	47 (82)

**Table 4:** Content validity indices

CTM-15		PACT-M1		PACT-M2	
Item no.	I-CVI	Item no.	I-CVI	Item no.	I-CVI
1	0.86	1	0.89	1	1.00
2	0.91	2	0.93	2	0.98
3	0.86	3	0.93	3	0.93
4	0.98	4	0.98	4	0.93
5	0.83	5	0.95	5	0.90
6	0.98	6	0.97	6	0.91
7	0.79	7	0.97	7	0.98
8	0.91	8	0.91	8	0.93
9	0.95				
10	0.86				
11	0.77				
12	0.95				
13	0.93				
14	0.95				
15	0.60				
Ave-CVI	0.88	Ave-CVI	0.94	Ave-CVI	0.95

Content validity index of each item (I-CVI) and the average content validity index (Ave-CVI) of the Care Transitions Measure (CTM-15), the Partners at Care Transitions Measure part 1 (PACT-M1), and the Partners at Care Transitions Measure part 2 (PACT-M2)

## Discussion

In this study we aimed to translate, cross-culturally adapt, and assess the content validity of the two questionnaires, CTM-15 and PACT-M for use in a Danish-speaking population. The results of pre-testing and cognitive interviewing indicated that both CTM-15 and the PACT-M questionnaires were relevant, comprehensive, and comprehensible to the patients.

The analysis of the quantitative data collected from the healthcare professionals i.e., the CVI computed at both the item and scale levels determined that PACT-M1 and PACT-M2 demonstrated excellent content validity among the healthcare professionals, whereas the CVI for two items of the CTM-15 fell below the threshold value for “good”, resulting in the ave-CVI falling just below the “acceptable” threshold of 0.9.

While there are some similarities between the CTM-15 and PACT-M as they both overall focus on the quality of the transition of care from the patient’s perspectives, there are also some differences. CTM-15 is widely recognised and used through the last two decades, while PACT-M is newly developed. CTM-15 captures the quality of the transition of care at one point of time, while PACT-M addresses both the immediate quality as well as coping at home within the first month after discharge. In this study, both instruments were well-received among the patients and healthcare professionals. However, a few concerns were noted.

The majority of respondents found the timing of CTM-15 inappropriate. The cognitive interviews were performed at the hospital on the day of discharge. As several items could only be answered after leaving the hospital, in future use, the CTM-15 should be administered after discharge to avoid these challenges. Another concern was that the CVI for item 11 from CTM-15, *“When I left the hospital, I was confident I could actually do the things I needed to do to take care of my health”*, at 0.77 fell just below the threshold value for “good”, indicating that healthcare professionals did not find this item highly relevant. Furthermore, item 15 from CTM-15 *“When I left the hospital, I clearly understood the possible****side effects****of each of my medications”* obtained a lower CVI of 0.60, potentially due to the perception among healthcare professionals that expecting patients to comprehend all side effects of every medication was not realistic.

Furthermore, some patients expressed concerns about the items related to homecare in the PACT-M questionnaires, as these specific items were not applicable to their situations. This could be due to the fact that we were validating the questionnaires on a target population with younger patients compared to the original target population, which was likely to result in a higher proportion of patients who did not need homecare. One possible approach to address these patients’ concerns could be to enhance the validation and utilisation of PACT-M by incorporating a “not applicable” response option for items 3, 4, and 8 in PACT-M2. This modification would ensure that the response options make sense also for patients who do not need homecare. When comparing across countries that do not provide this response option, the “not applicable”-responses can be added to the “strongly agree”-responses, as suggested by the developers.

Although the study was performed on patients with multimorbidity who were admitted to the hospital due to an acute medical condition, the results may be applicable to other similar populations e.g., patients with multimorbidity who are admitted due to non-medical conditions, or patients with less than two chronic conditions.

The study demonstrates several strengths that contribute to the credibility of the findings. Firstly, there is a strong variation of patient demographics in both Patient Panel I and Patient Panel II, increasing the likelihood that the results are useful in the target population. Secondly, the participation of healthcare professionals from all sectors of the Danish healthcare system ensures that the included items are important to clinicians, and are considered relevant and comprehensive for assessing the intended construct. Furthermore, the interviews were conducted systematically, ensuring consistency in data collection. The analyses were also approached systematically, enhancing the rigor of the study. Additionally, the inclusion of both quantitative and qualitative data provides a comprehensive evaluation of the questionnaires.

However, certain limitations should be acknowledged. One limitation is the limited participation of municipal healthcare professionals. They are primarily responsible for patients after discharge, and more responses from municipal healthcare professionals would have added further strength to the validation. The timing of the cognitive interviews regarding CTM-15 can also be considered a limitation, as many items in the questionnaire referred to activities that had not yet taken place, making it challenging for the patients to evaluate the relevance of the items.

## Conclusion

In this study we have developed Danish versions of the CTM-15, PACT-M1, and PACT-M2. Minor cultural adaptations were made during the translation procedure to ensure the questionnaires are suitable for adult patients with multimorbidity in the Danish healthcare system.

Furthermore, we have investigated the relevance, comprehensibility, and comprehensiveness of the questionnaires, and the findings indicate that both questionnaires effectively measure the intended construct, establishing their content validity in a Danish context.

The PACT-M questionnaires showed good content validity among patients as well as among healthcare professionals. However, we suggest adding the “not applicable” answer option in PACT-M2 items 3, 4 and 8 in the further validation process in a Danish context.

Overall, we consider the content validity of CTM-15 to be acceptable, given that patients found it relevant, comprehensible and comprehensive, and only two items achieved a low I-CVI from the healthcare professionals. Consequently, we recommend retaining all items in CTM-15 and allowing ongoing validation processes to determine whether a shortened version of CTM-15 would be more suitable in a Danish context.

Further validation of both CTM-15 and PACT-M in order to investigate their construct validity, reliability, and responsiveness is needed.

The fully validated instruments will have the potential to provide valuable insights into the patients’ experiences of the transition of care. This will be highly relevant in future research projects aiming at investigating and improving transition of care. Additionally, the instruments can be utilised to identify target groups for interventions aiming at transition of care, quality monitoring, or improvement projects.

## Data Availability

The data described in this article is not publicly available in further detail beyond that provided in the manuscript.
